# Knowledge Gaps in the Pharmacokinetics of Therapeutic Proteins in Pediatric Patients

**DOI:** 10.3389/fphar.2022.847021

**Published:** 2022-02-10

**Authors:** Bernd Meibohm

**Affiliations:** Department of Pharmaceutical Sciences, College of Pharmacy, The University of Tennessee Health Science Center, Memphis, TN, United States

**Keywords:** pediatrics, therapeutic proteins, pharmacokinetics, pediatric extrapolation, ontogeny, neonatal Fc receptor, mannose receptor, asialoglycoprotein receptor

## Abstract

Therapeutic proteins such as monoclonal antibodies and their derivatives, fusions proteins, hormone analogs and enzymes for replacement therapy are an ever-growing mainstay in our pharmacopoeia. While a growing number of these medications are developed for and used in younger and younger pediatric patients, knowledge gaps in the basic understanding of the molecular and physiologic processes governing the disposition of these compounds in the human body and their modulation by age and childhood development are a hindrance to the effective and timely development and clinical use of these compounds, especially in very young pediatric patient populations. This is particularly the case for the widespread lack of information on the ontogeny and age-associated expression and function of receptor systems that are involved in the molecular processes driving the pharmacokinetics of these compounds. This article briefly highlights three receptor systems as examples, the neonatal Fc receptor, the asialoglycoprotein receptor, and the mannose receptor. It furthermore provides suggestions on how these gaps should be addressed and prioritized to provide the field of pediatric clinical pharmacology the urgently needed tools for a more effective development and clinical utilization of this important class of drugs with rapidly evolving importance as cornerstone in pediatric pharmacotherapy.

## Introduction

Over the past 25 years, therapeutic proteins such as monoclonal antibodies (mAbs) and their derivatives, fusions proteins, hormone analogs and enzymes for replacement therapy have gained major roles in the armamentarium to treat numerous conditions and diseases (Crommelin et al., 2019). More recently, constructs that are the result of advanced protein engineering such as bispecifics and similar innovative molecules have been added to this group of molecules and are receiving major attention in drug development programs (Rathi and Meibohm, 2015; Brinkmann and Kontermann, 2017). While these protein-based medications are typically first developed and approved for adult patient populations, extensions of regulatory approval for use in pediatric populations is frequently pursued after initial market introduction (Zhang et al., 2015; Temrikar et al., 2020). These efforts have been further spurred and formalized by regulatory incentives and regulatory requirements that have been established over the past 3 decades in Europe and North America (Zisowsky et al., 2010; Zhang et al., 2015). In this context, there is an ever growing need to establish dosage regimens and dosing recommendations that address the specific needs of different age groups of pediatric patients to ensure a safe and effective pharmacotherapy in these patient populations (Xu et al., 2013; Malik et al., 2021).

While pediatric dosing may be affected by differences in pharmacokinetic as well as pharmacodynamic processes but also differences in disease etiology and progression, particular interest has often been directed towards pharmacokinetic differences. This is based on the notion that full and partial extrapolation approaches of efficacy from adults to children frequently rely on exposure-matching where dosing regimens of the drug in question in different pediatric populations are selected in such a way that they achieve drug exposures in the pediatric patients “similar” to those having shown to be efficacious and safe in adults (Mulugeta et al., 2016). This approach of course relies on the assumption that the course of the disease and the response to the drug are sufficiently similar between adults and the considered pediatric population, a prerequisite that needs to be supported by adequate data.

## Key Mechanisms of Drug Disposition Processes for Therapeutic Proteins

The pharmacokinetic processes of distribution and elimination of therapeutic proteins are governed by combinations of physicochemical, physiologic and receptor-mediated processes and have been reviewed in detail elsewhere (Tang et al., 2004; Mould and Meibohm, 2016; Ryman and Meibohm, 2017; Meibohm, 2019). In brief, distribution is largely determined by molecule size and charge. Large therapeutic proteins such as mAbs with a molecular weight of 150 kDa are largely confined to the vascular space with only limited distribution into the interstitial space of extravascular organs and tissues. Distribution for these molecules is largely driven by convective extravasation that is, determined by the number and size of pores between endothelial cells lining the blood and lymphatic vessels and pressure gradients between hydrostatic and colloid osmotic pressure in vascular, interstitial and lymphatic spaces and capillaries.

Elimination processes can broadly be distinguished into unspecific proteolytic degradation that can either be receptor-mediated or non-receptor-mediated (Meibohm, 2019). Non-receptor-mediated processes are usually initiated by pinocytosis, a fluid-phase endocytotic cellular uptake of the therapeutic protein molecule followed by intracellular lysosomal degradation to small peptides and amino acids. This degradation process is performed by phagocytic cells of the reticuloendothelial system as well as endothelial cells lining blood and lymph capillaries. Organs with major capillary beds such as muscle, skin and to lesser degree the intestine as well as organs with high number of phagocytic cells are thus major contributors to this nonspecific proteolytic degradation (Eigenmann et al., 2017). In case of receptor-mediated proteolysis, the intracellular uptake may be mediated by membrane-standing promiscuous receptor systems, for example, the LDL-receptor, or sugar-recognizing receptors such as the mannose receptor. Usually, receptor-mediated uptake processes are substantially faster and more efficient than pinocytosis, and proteins using these pathways are more rapidly eliminated. In the specific case where the membrane receptor that facilitates the intracellular uptake is the pharmacologic target, one refers to target-mediated elimination. Due to the usually high binding affinity of the therapeutic protein for its pharmacologic target, the target-mediated elimination process is usually substantially faster than the elimination processes relying on pinocytosis or “unspecific” receptor-mediated endocytosis (Tang et al., 2004). For mAbs and antibody-derivatives, interaction with immunoglobulin-specific receptors such as the neonatal Fc-receptor (FcRn) and Fcγ receptors may also affect the clearance of these therapeutic proteins. Interaction with FcRn in the acidified lysosome after intracellular uptake may prevent IgG molecules and thus mAbs from proteolytic degradation, thereby leading to an increased residence time and thus decreased clearance of these molecules in the systemic circulation (Ryman and Meibohm, 2017). Interaction between mAbs and Fcγ-receptors expressed on immune cells, while highly relevant for processing and removal of immune complexes, may constitute additional elimination pathways, although their overall contribution seems to be limited for the majority of mAbs (Thomas and Balthasar, 2019). For small therapeutic proteins below the glomerular filtration cutoff of approximately 60 kDa, proteolytic degradation in proximal tubular cells after glomerular filtration in the kidneys may also contribute to their clearance (Meibohm and Zhou, 2012).

## Differences in Therapeutic Protein Disposition Between Children and Adults and Related Knowledge Gaps

Pediatric extrapolation efforts to establish dosing regimens for therapeutic proteins are hampered by a lack of a comprehensive understanding of the differences in drug distribution and elimination mechanisms between children and adults, particularly young pediatric patients such as full term and premature neonates and infants, i.e., in the range younger than 1 year of age. While many disposition processes based on physicochemical and physiologic processes are reasonably well understood, those related to receptor-mediated processes remain in many instances unclear or elusive. In more general terms, size-related differences between children and adults have relatively well been characterized, while knowledge on pediatric maturation-related differences remains spotty.

The distribution processes of most therapeutic proteins, as described in the previous section, are largely driven by conserved physicochemical processes together with physiologic differences between adults and different pediatric age groups and can thus usually be well predicted for pediatric populations. Therefore, allometric scaling approaches accounting for body size differences between children and adults usually characterize the distribution of therapeutic proteins well. Only for very young pediatric patients such as newborns and infants, further differences may need to be considered. These include the well-known higher total and extracellular tissue water content, larger capillary beds and thus capillary surface area per tissue volume, and higher perfusion rates (Friis-Hansen, 1983; Malik and Edginton, 2018). All these processes together would be expected to result in faster extravasation of therapeutic proteins, lower concentration differences between the vascular and the extravascular space, and overall larger extravascular distribution volumes per volume unit of tissue (Temrikar et al., 2020). While an allometric exponent of 1 has widely been used to scale distribution volumes between children and adults based on body weight (Meibohm et al., 2005), more recent analyses considering a diverse set of protein-based therapeutics suggest that an exponent of 0.8 might be more appropriate (Malik et al., 2021).

Similar to distribution volumes, clearance values for non-receptor-mediated proteolytic degradation processes of therapeutic proteins in children can also relatively well be derived from adult values based on allometric scaling with allometric exponents of 0.75 or 0.85 accounting solely for body size-based differences between children and adults (Malik et al., 2021). Only for children younger than 1 year of age, maturation-related differences also have to be considered. For example, young infants, newborns and particularly low-birth weight infants have been reported to exhibit a 2–3 times higher lysosomal protein turnover normalized for body weight (Beaufrere, 1994), which would be expected to affect unspecific proteolytic degradation and result in an increased protein clearance (Temrikar et al., 2020).

For receptor-mediated elimination processes, however, the available knowledgebase on age- and maturation-related differences between children and adults is very scarce. For FcRn, for example, data have been limited to rodent studies. While messenger RNA (mRNA) expression of the α-chain of FcRn in rats suggested an age-associated increase (Tian et al., 2014), more recent results on age-associated expression at the protein level in mice suggest no relevant differences in expression from newborn through juvenile animals to adults in skin and spleen tissues (Limothai, 2015), which may be interpreted as more definitive due to the often limited mRNA-to-functional protein correlation for many endogenous proteins including FcRn (Li and Balthasar, 2018; Temrikar et al., 2020). There are currently no human data yet available on the ontogeny of FcRn, especially in very young pediatric patients. A more likely age-associated effect on FcRn recycling of mAbs and their derivatives are the well documented substantially lower reference values for endogenous IgG subclasses in infants compared to older children and adults (Plebani et al., 1989) that would be expected to lead to less endogenous competition for FcRn and thus a more efficient recycling process with potentially reduced clearance for protein molecules interacting with FcRn (Temrikar et al., 2020).

An example for a promiscuous membrane receptor facilitating the uptake of therapeutic proteins for subsequent lysosomal degradation is the asialoglycoprotein receptor (ASGPR) (Stockert, 1995). It is expressed on hepatocytes and facilitates the uptake of proteins that carry a glycan chain with a terminal galactose or galactose derivative. Examples are erythropoietin, reteplase, lanoteplase and clotting factor VIII (Lunghi et al., 2021). ASGPR has also been implicated in the glycoform selective clearance of therapeutic proteins with complex N- or O-linked glycosylation structures (Jones et al., 2007; Stefanich et al., 2008). More recently, ASGPR has also been utilized to facilitate hepatic targeting of N-acetylgalactosamine-conjugated RNA interference therapeutics (Li et al., 2021). Data on ASGPR expression and activity in children is very limited. While ASGPR has been detected in human fetal liver (Yoshida et al., 1999), age-related expression levels are limited to mice where activity increased postpartum and reached adult levels after 5 days (Collins et al., 1984). Additional knowledge has been inferred by physiologic pharmacokinetic modelling of pharmacokinetic data for known ASGPR substrates from different species (Poulin, 2011).

Similar to ASGPR, the mannose receptor is a highly effective endocytic receptor that is expressed on selected populations of macrophages and dendritic cells, and that recognizes glycoproteins with mannosylated glycan chains (Martinez-Pomares, 2012). High-mannose glycoforms of mAbs have increased clearance compared to mAbs with other glycans due to interaction with the mannose receptor (Falck et al., 2021). The age-associated expression of the mannose receptor is largely unknown. In mice, the mannose receptor was first detected on macrophages on day 10 in the embryonic stage and persisted postnatally thereafter (Takahashi et al., 1998). This may imply that mannose receptor activity has already reached adult levels at birth. The major role of FcRn, ASGPR, and the mannose receptor om the disposition of therapeutic proteins are summarized in Table 1.

**TABLE 1:** Examples of receptor systems affecting the pharmacokinetics of therapeutic proteins with unknown ontogeny.

Receptor system	Tissues with high expression	Recognized molecular structure	Examples for affected therapeutic proteins
Asialoglycoprotein receptor (ASGPR)	Hepatocytes (sinusoidal surface)	Glycan chains with terminal galactose or N-acetylgalactosamine residues	Erythropoietin; FSH; clotting factors VII, VIII, IX; reteplase, lanoteplase
Mannose receptor	Macrophages, immature dendritic cells, and liver sinusoidal endothelial cells	Glycan chains with high mannose content (M5-M9)	High mannose forms for IgG monoclonal antibodies and their derivatives
Neonatal Fc receptor (FcRn)	Vascular endothelial cells and phagocytic cells as well as other cell types, particularly in liver, spleen, intestine, lungs and lymph nodes	FcRn pH-dependent binding site on the constant domain of IgG molecule and albumin	Monoclonal antibodies; antibody-derivatives and fusion proteins with intact FcRn-binding site on the Fc domain; albumin fusion proteins

For target-mediated drug disposition processes, data are even more scarce than for those elimination processes related to less specific receptor systems such as ASGPR or the mannose receptor. One might expect that each target has its own specific ontogeny with age- and maturation-associated differences in target receptor abundance, turnover and internalization kinetics (Temrikar et al., 2020). This becomes especially challenging when new therapeutic targets and/or novel indications are pursued. In addition, individual pediatric patients usually follow different temporal developmental trajectories that further complicate and individualize their dose requirements for a specific therapeutic protein (Barrett et al., 2012).

## Discussion and Perspectives

The selection of safe and efficacious dosing regimens for drug development and applied pharmacotherapy of therapeutic proteins in pediatric patients is severely hampered by substantial knowledge gaps on the ontogeny and age-associated expression and function of receptor systems that are involved in the molecular processes driving the pharmacokinetics of these compounds. This is particularly relevant for newborns and infants where differences in therapeutic protein pharmacokinetics cannot be fully explained by size differences between children and adults and where additional maturation processes need to be considered. This article briefly highlighted three receptor systems as examples, FcRn, ASGPR and the mannose receptor, but numerous others may be involved in the disposition process of specific therapeutic proteins as well. Priorities for filling these knowledge gaps should be initially directed towards those receptor systems that are more broadly relevant to the largest number of therapeutic proteins, for example, FcRn for all mAbs and mAb derivatives with intact FcRn binding site.

Population pharmacokinetic modeling (PopPK) and physiological pharmacokinetic modeling (PBPK) have been widely used in support of pediatric extrapolation exercises based on exposure-matching approaches for traditional small molecule drugs (Conklin et al., 2019). While PopPK is a data-driven, deductive modeling approach, PBPK can be viewed as an inductive approach based on the integrated prior knowledge of drug- and system-specific parameters and structures (Barrett et al., 2012). A recent analysis of FDA approval data for the 20 monoclonal antibodies and Fc-fusion proteins approved at the time in both adult and pediatric indications revealed that while 19 of the 20 projects included PopPK based modeling and simulation in support of the selected pediatric dosing regimens, only one of them included a PBPK approach (Liu et al., 2019). This lack of use of PBPK for therapeutic proteins in pediatric indications may partially be related to the highlighted knowledge gaps in understanding pediatric disposition of these molecules as PBPK rather than PopPK is largely dependent on an intrinsic understanding of the drug disposition mechanisms and pathways that underlie a therapeutic protein’s pharmacokinetic behavior.

There have recently been elegant attempts to impute the lack of age-associated function of receptor systems such as FcRn through PBPK modeling frameworks using known PK data of endogenous and therapeutic proteins (Hardiansyah and Ng, 2018; Pan et al., 2020). While these approaches are pragmatic in the current situation, they still cannot overcome the residual uncertainty associated with the arbitrary assignment of age-associated disposition behavior to one unmeasured model component. This underlines the need for basic molecular pharmacology investigations in the age groups of interest to fill our existing knowledge gaps with high quality data. The gained knowledge would likely not only benefit one specific development project or compound but would likely be more broadly applicable. These opportunities to add to the collective pediatric drug disposition knowledgebase will be crucial to advance the reliability and reduce the uncertainty of pediatric extrapolation efforts (Laer et al., 2009; Temrikar et al., 2020). Only then will the currently existing uncertainties in extrapolation of therapeutic proteins to pediatric patients be overcome and a more widespread application of prospective modeling frameworks in this area be possible.

## Data Availability

The original contributions presented in the study are included in the article/supplementary material, further inquiries can be directed to the corresponding author.
